# Effects of propofol and its formulation components on macrophages and neutrophils in obese and lean animals

**DOI:** 10.1002/prp2.873

**Published:** 2021-10-10

**Authors:** Luciana Boavista Barros Heil, Fernanda Ferreira Cruz, Mariana Alves Antunes, Cassia Lisboa Braga, Lais Costa Agra, Rebecca Madureira Bose Leão, Soraia Carvalho Abreu, Paolo Pelosi, Pedro Leme Silva, Patricia Rieken Macedo Rocco

**Affiliations:** ^1^ Laboratory of Pulmonary Investigation Carlos Chagas Filho Institute of Biophysics Federal University of Rio de Janeiro Rio de Janeiro Brazil; ^2^ Department of Surgical Sciences and Integrated Diagnostics University of Genoa Genoa Italy; ^3^ Anesthesia and Intensive Care San Martino Policlinico Hospital – IRCCS for Oncology and Neurosciences University of Genoa Genoa Italy

**Keywords:** adipose tissue, inflammation, lung, macrophages, neutrophils, obesity, propofol

## Abstract

We hypothesized whether propofol or active propofol component (2,6‐diisopropylphenol [DIPPH] and lipid excipient [LIP‐EXC]) separately may alter inflammatory mediators expressed by macrophages and neutrophils in lean and obese rats. Male Wistar rats (*n* = 10) were randomly assigned to receive a standard (lean) or obesity‐inducing diet (obese) for 12 weeks. Animals were euthanized, and alveolar macrophages and neutrophils from lean and obese animals were exposed to propofol (50 µM), active propofol component (50 µM, 2,6‐DIPPH), and lipid excipient (soybean oil, purified egg phospholipid, and glycerol) for 1 h. The primary outcome was IL‐6 expression after propofol and its components exposure by alveolar macrophages extracted from bronchoalveolar lavage fluid. The secondary outcomes were the production of mediators released by macrophages from adipose tissue, and neutrophils from lung and adipose tissues, and neutrophil migration. IL‐6 increased after the exposure to both propofol (median [interquartile range] 4.14[1.95–5.20]; *p* = .04) and its active component (2,6‐DIPPH) (4.09[1.67–5.91]; *p* = .04) in alveolar macrophages from obese animals. However, only 2,6‐DIPPH increased IL‐10 expression (7.59[6.28–12.95]; *p* = .001) in adipose tissue‐derived macrophages. Additionally, 2,6‐DIPPH increased C‐X‐C chemokine receptor 2 and 4 (CXCR2 and CXCR4, respectively) in lung (10.08[8.23–29.01]; *p* = .02; 1.55[1.49–3.43]; *p* = .02) and adipose tissues (8.78[4.15–11.57]; *p* = .03; 2.86[2.17–3.71]; *p* = .01), as well as improved lung‐derived neutrophil migration (28.00[−3.42 to 45.07]; *p* = .001). In obesity, the active component of propofol affected both the M1 and M2 markers as well as neutrophils in both alveolar and adipose tissue cells, suggesting that lipid excipient may hinder the effects of active propofol.

## Graphical abstract

In obesity, the active component of propofol affected M1 and M2 alveolar macrophages from adipose tissue differently. In neutrophils from lung and adipose tissue, only the active propofol component increased chemokine receptors, suggesting that lipid excipient may hinder the effects of active propofol.

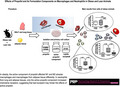

**Abbreviations**
2,6‐DIPPH: 2,6‐diisopropylphenol
2,6‐DIPPH +LIP‐EXC: propofol composition
CXCR‐2: CXC chemokine receptor 2
CXCR‐4: CXC chemokine receptor 4
DMEM: Dulbecco's modified Eagle's medium
ECM: extracellular matrix
EDTA: ethylenediamine tetraacetic acid
FBS: fetal bovine serum
ICAM‐1: intracellular cell adhesion molecule 1
IL: interleukin
IMDM: Iscove's modified Dulbecco's medium
iNOS: inducible nitric oxide synthase
LCT: long‐chain triglyceride
LIP‐EXC: lipid excipient
PBS: phosphate‐buffered saline
PC‐I: procollagen type I
PC‐III: procollagen type III
PCR: polymerase chain reaction
RT: reverse transcription
SO: soybean oil
TGF‐β: transforming growth factor beta
VCAM‐1: vascular cell adhesion molecule 1

## 1 INTRODUCTION

Obesity is a complex multifactorial disease associated with several comorbidities.^1^ The chronic low‐grade inflammatory condition of obesity orchestrated by immune cell disruption has relevant negative consequences.^2^, ^3^ Anesthesia and the perioperative stress response can modulate several immunological reactions, which are very diverse and still not fully understood in obesity. The immunomodulatory effects of anesthetic agents have been observed in clinical^4^, ^5^ and pre‐clinical^6^, ^7^ studies involving the perioperative period and models of acute inflammation that were distinct from the low‐grade systemic inflammation in obesity.

Propofol, a widely used anesthetic agent, is composed of its active component (2,6‐diisopropylphenol [DIPPH]) and lipid excipient (LIP‐EXC) (soybean oil, glycerol, and egg lecithin), which may have different biological activities.^8^ Propofol immunomodulatory properties have been investigated in several models of inflammation, and that might have had an impact on perioperative outcomes, depending on the context, favoring pro‐ or anti‐inflammatory responses. *In vitro* studies, propofol decreases chemotaxis, phagocytic activities, oxidative ability,^9^ and the release of inflammatory mediators^10^ by reducing nuclear factor (NF)‐κB activation.^11^ In neutrophils, propofol composition is also capable of inhibiting elastase release.^12^ Recently, in experimental obesity, controversial results that may be associated with the composition of propofol yielded increased airway resistance and interleukin (IL)‐6 levels.^13^ These results have led to questions regarding the mechanisms of propofol composition or its main formulation components in obesity‐related inflammation. Within this context, we hypothesized that both active propofol components, DIPPH and LIP‐EXC, may have different impacts on inflammatory mediators expressed by macrophages, neutrophils, and other structural cells in obesity. The primary outcome was to assess changes in IL‐6 expression induced by propofol and its components in alveolar macrophages extracted from bronchoalveolar lavage fluid. The secondary outcomes were to analyze the production of mediators from lung tissue, neutrophil migration, mediators associated with vascular adhesion in endothelial cells, and fibrogenesis in fibroblasts. Moreover, mediators released by macrophages and neutrophils were also evaluated in adipose tissue.

## 2 METHODS

### 2.1 Study approval

This study was approved by the Ethics Committee of the Health Sciences Center of Federal University of Rio de Janeiro (020/16 CEUA‐UFRJ Rio de Janeiro, Brazil). All animals received humane care in compliance with the Principles of Laboratory Animal Care formulated by the National Society for Medical Research and the Guide for the Care and Use of Laboratory Animals prepared by the US National Academy of Sciences. Animals experiments followed the ARRIVE guidelines.^14^ Non‐specific‐pathogen‐free animals were housed in a controlled temperature (23°C) and controlled light–dark cycle (12–12 h), with free access to water and food, with acclimation for 1 week.

### 2.2 Animal preparation and experimental protocol

Healthy male Wistar rats (*n* = 10, weight 130 ± 30 g, age 5 weeks) were randomly assigned to receive a conventional diet (commercial rodent chow, macronutrients; proteins: 22% kcal; lipids: 10% kcal; carbohydrates: 68% kcal; calories: 3.9 kcal/g; Purina, São Paulo, Brazil) (lean group, *n* = 5) and an obesity‐inducing diet, developed by supplementing the conventional diet with a tablet, a confectionery product made from sweetened condensed milk (obese experimental diet, macronutrients; proteins: 14% kcal; lipids: 13% kcal; carbohydrates: 73% kcal; calories: 4.0 kcal/g)^13^ (obese group, *n* = 5) for 12 weeks.^13^ After induction of obesity, animals were euthanized by a lethal dose (150 mg/kg intraperitoneally) of sodium thiopental (Thiopentax, Cristália, Itapira, São Paulo, Brazil). All procedures for cell extraction and isolation were similar in both lean and obese groups.

### 2.3 Bronchoalveolar lavage

Alveolar macrophages were collected from lungs by bronchoalveolar lavage. A polyethylene cannula was inserted into the trachea and a total volume of 1.5 ml of warm phosphate‐buffered saline (PBS) containing 10 mM ethylenediamine tetraacetic acid (EDTA) at 37°C was instilled and the lungs washed three times. Samples were centrifuged at 300× *g* for 5 min at 4°C. The supernatant was removed, and cells in the pellet were resuspended in 1× PBS.

To ensure enough cells for assay, alveolar macrophages from each group were pooled and pressed through a 40‐μm mesh filter into a single‐cell suspension. Cells were then cultured in a six‐well culture plate at 37°C with 5% CO_2_ at a concentration of 10^5^ cells per well in 2 ml of Roswell Park Memorial Institute 1640 medium (Sigma Chemical Co) supplemented with 10% fetal bovine serum (FBS) (Invitrogen), 2 mM L‐glutamine (Life Technologies), 100 U/ml penicillin, and 0.1 mg/ml streptomycin.^15^ After incubation for 2 h, non‐adherent cells were washed off with PBS, and the medium was replaced.

### 2.4 Isolation of lung cells

Lungs were removed and the left lung was used to isolate neutrophils, endothelial cells, and fibroblasts.

#### 2.4.1 Lung neutrophils

Lung neutrophils were isolated according to the standard method of sedimentation, centrifugation in Hystopaque density gradient separation followed by hypotonic lysis of red blood cells.^12^ The purified neutrophils were immediately submitted to drug exposure.

#### 2.4.2 Lung endothelial cells

The left lung was cut into small pieces and digested with collagenase 1% for 40 min under mild agitation. Iscove's modified Dulbecco's medium (IMDM) (Invitrogen Life Technologies) supplemented with 10% FBS and 1% penicillin/streptomycin was added to the collagenase solution, and the sample was strained through 70‐µm cell strainers. Samples were washed with PBS twice to remove serum. The tube was then centrifuged at 300× *g* for 5 min, and the pellet resuspended and incubated in 100 μl of PBS containing 2 μl of anti‐CD54 antibody (1:100, catalog no. B183252, BioLegend) for 20 min. Samples were then washed with PBS and centrifuged at 300× *g* for 5 min. Cells were resuspended in PBS and then incubated with Dynabeads biotin binder (Thermo Fisher) according to the manufacturer's instructions for 20 min on ice. After incubation, cells were isolated by exposure to a magnetic field. Finally, the pellet was suspended with IMDM supplemented with 10% calf serum (Hyclone), 1% penicillin/streptomycin, and 1% glutamine (Invitrogen Life Technologies), and transferred to six‐well plates pre‐coated with 0.1% gelatin.^16^

#### 2.4.3 Lung fibroblasts

Lung tissue was cut into small pieces and subjected to enzymatic collagenase digestion for 45–60 min at 37°C. The reaction was blocked by adding Dulbecco's modified Eagle's medium (DMEM) with 10% FBS and immediately centrifuged at 300× *g* for 5 min. Cells were plated in DMEM‐F12, 10% FBS, 1000 U/ml penicillin/streptomycin antibiotic solution, and 2 mM L‐glutamine and kept in a humidified chamber with 5% CO_2_ at 37°C.

### 2.5 Isolation of macrophages and neutrophils in adipose tissue

Epididymal fat pad tissue was collected, rinsed in PBS, transferred to a Petri dish, and cut into small pieces. The dissected pieces (around 0.2–0.8 cm^3^) were washed with PBS, cut into smaller fragments (1 mm), and subsequently digested with collagenase type I (1 mg/ml) with agitation for 40 min at 37°C. Proteolysis was stopped with supplemented media, centrifuged, the pellet was resuspended, and then macrophages and neutrophils were isolated through a Percoll gradient solution.^17^

### 2.6 Cell culture

Macrophages, fibroblasts, and endothelial cells were incubated at 37°C in a humidified atmosphere chamber with 5% CO_2_, 21% O_2_, and 74% N_2_. The supernatant was discarded, non‐adherent cells were removed, and specific fresh culture medium was changed every 2 days. Adherent cells reaching 80% confluence were lifted with trypsin‐EDTA solution (Gibco, Rockville, MD, USA) and then used in further experiments.

### 2.7 Drug exposure

First passage cells were plated in six‐well plates (approximately 1 × 10^6^ cells per well) for 48 h. The medium was replaced with fresh medium and cells were exposed to 50 µM propofol (2,6‐DIPPH + LIP‐EXC; Cristália Laboratories),^10^ or its components: (1) active propofol (2,6‐DIPPH; Sigma‐Aldrich) and (2) lipid excipient (LIP‐EXC; Cristália Laboratories) for 1 h. Cells were then washed with 1× PBS, lifted using 2.5% trypsin/EDTA, and pelleted by centrifugation (300× *g* for 5 min) for further analysis.

### 2.8 Biological markers

A quantitative real‐time reverse transcription (RT) polymerase chain reaction (PCR) was done to measure the mRNA expression of biological markers associated with (1) macrophage polarization, such as pro‐inflammatory (M1 markers) interleukin (IL)‐6 and inducible nitric oxide synthase (iNOS) and anti‐inflammatory (M2 markers) IL‐10, transforming growth factor (TGF)‐β, and arginase; (2) neutrophil trafficking to the lung, the CXC chemokine receptors (CXCR‐2 and CXCR‐4); (3) fibrogenesis, procollagen (PC)‐I, procollagen PC‐III, and TGF‐β; and (4) endothelial cell damage and vascular cell adhesion molecule (VCAM)‐1.

Cells were lysed for RNA extraction with ReliaPrep RNA Tissue Miniprep System (Promega) according to the manufacturer's instructions. The total RNA concentration was measured by spectrophotometry in a Nanodrop ND‐2000 system. First‐strand cDNA was synthesized from total RNA using a High‐Capacity cDNA Reverse Transcription Kit (Thermo Fisher). Real‐time PCR primers for the target gene were purchased from Invitrogen. Relative mRNA levels were measured with the SYBR Green system (Promega) using a real‐time PCR Realplex Mastercycler (Eppendorf). All samples were measured in triplicate. The relative expression of each gene was calculated as a ratio of the gene of interest to the control gene (acidic ribosomal phosphoprotein P0, *36B4*) and expressed as fold change relative to cells from the obese or lean group treated with saline (2^−ΔΔCt^).^18^ The primer sequences are listed in Table 1.

**TABLE 1:** Forward and reverse oligonucleotide sequences of target gene primers used in the experiments

Gene	Primer	Primer sequences (5′‐3′)
IL‐6	Forward	CTCCGCAAGAGACTTCCAG
	Reverse	CTC CTCTCCGGACTTGTGA
iNOS	Forward	CTTCAGGTATGCGGTATTGG
	Reverse	CATGGTGAACACGTTCTTGG
IL‐10	Forward	AGAAGGACCAGCTGGACAAC
	Reverse	GTCGCAGCTGTATCCAGAGG
TGF‐β	Forward	ATACGCCTGAGTGGCTGTC
	Reverse	GCCCTG TATTCCGTCTCCT
Arginase	Forward	TTGATGTTGATGGACTGGAC
	Reverse	TCTCTGGCTTATGATTACCTTC
CXCR2	Forward	ATCTTTGCTGTGGTCCTCGT
	Reverse	GGGGTTAAGACAGCTGTGGA
CXCR4	Forward	CTCTGAGGCGTTTGGTGCT
	Reverse	TGCCCACTATGCCAGTCAAG
Procollagen I (PC‐I)	Forward	AGAAGTCTCAAGATGGTGGCCG
	Reverse	GGTCACGAACCACGTTAGCATC
Procollagen III (PC‐III)	Forward	CAGCTATGGCCCTCCTGA TCTT
	Reverse	GTAATGTTCTGGGAGGCCCG
VCAM	Forward	TGCACGGTCCCTAATGTGTA
	Reverse	TGCCAATTTCCTCCCTTAAA
*36B4*	Forward	AATCCTGAGCGATGTGCAG
	Reverse	GCTGCCATTGTCAAACAC

Abbreviations: CXCR2, chemokine CXC receptor 2; CXCR4, chemokine CXC receptor 4; IL‐6, interleukin‐6; IL‐10, interleukin‐10; iNOS, inducible nitric oxide; TGF‐β, transforming growth factor beta; *36B4*, acidic ribosomal phosphoprotein P0.

### 2.9 Neutrophil migration assay

Fresh lung and adipose tissue neutrophils from obese animals, obtained as described above, were either stimulated with (1) regular medium (0.9% sodium chloride); (2) 50 μM propofol (2,6‐DIPPH + LIP‐EXC), (3) active propofol component (2,6‐DIPPH, Sigma‐Aldrich); or (4) the lipid excipient (LIP‐EXC, Cristália Laboratories) for 1 h. Cells were seeded on the upper chamber of 3 μm pore membrane transwell chambers (Capsule Cups, Cytosensor Microphysiometer, Molecular Devices) in a 12‐well plate for 1 h. The bottom chamber was filled with culture medium alone or in the presence of cytokine‐induced neutrophil attracting chemokine (0.1 μg/ml).^19^

To determine the percentage of neutrophils that had migrated to the bottom chamber, a light microscope (Olympus) and a Neubauer chamber were used to count the number of cells that were able to migrate from the upper toward the bottom well, compared with the amount of cells that were seeded. Data represent the percentage variation related to regular medium (0.9% sodium chloride) from three independent experiments for each treatment condition.

### 2.10 Statistical analysis

A sample size of 10 animals (five per group between lean and obese animals) would provide the appropriate power (1 − β = 0.8) to identify significant (α = 0.05) differences in IL‐6 gene expression in alveolar macrophages treated with propofol for 1 h^10^ taking into account effect size (*d* = 2.29), a two‐sided test, and a sample size ratio of 1 (G*power 3.1.9.2; University of Dusseldorf, Dusseldorf, Germany). Data were tested for normality using the Kolmogorov–Smirnov test with Lilliefors’ correction, and the Levene median test was used to evaluate homogeneity of variances.

For nonparametric data, the Kruskal–Wallis test followed by Dunn's multiple comparisons test was used, and the data are expressed as medians (interquartile range). All analyses were performed in a blinded manner (i.e., the observer was unaware of the experimental protocol) using the Prism version 8.1.1 software package (GraphPad Software; http://www.graphpad.com), and statistical significance was established at *p* < .05.

## 3 RESULTS

### 3.1 Cells isolated from obese animals

#### 3.1.1 Effects of propofol (2,6‐DIPPH + LIP‐EXC), the active propofol component (2,6‐DIPPH), and lipid excipient (LIP‐EXC) on alveolar and adipose tissue macrophages

**FIGURE 1:**
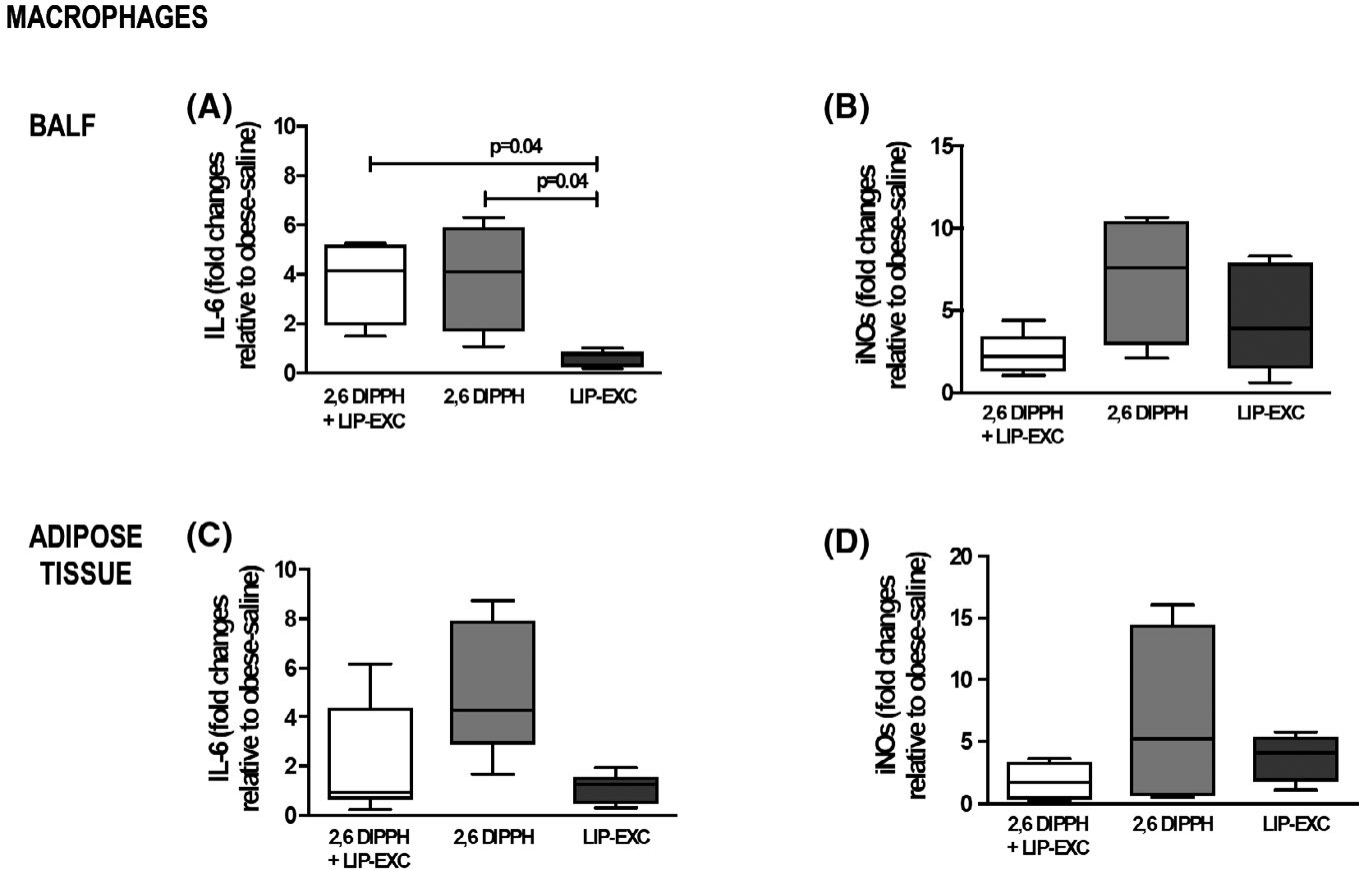
Alveolar and adipose tissue macrophages from obese animals (*n* = 5). Gene expression of interleukin (IL)‐6 (A, C) and inducible nitric oxide synthase (iNOS) (B, D) was assessed by reverse transcription polymerase chain reaction in macrophages stimulated for 1 h with propofol composition (2,6‐diisopropylphenol [2,6‐DIPPH] + lipid excipient [LIP‐EXC]), active propofol component (2,6‐DIPPH), or LIP‐EXC. Data represent relative gene expression calculated as a ratio of the average expression of the target gene compared with the reference gene *36B4* and expressed as fold change relative to unstimulated cells. Samples were measured in triplicate; values are depicted as box plots (median, interquartile range, and minimum and maximum). Comparisons between groups were performed using the Kruskal–Wallis test followed by Dunn's post‐hoc test. BALF, bronchoalveolar lavage fluid

In alveolar macrophages, IL‐6 expression was higher after therapy with 2,6‐DIPPH + LIP‐EXC (median [interquartile range], 4.14 [1.95–5.20]; *p* = .04) and 2,6‐DIPPH (4.09 [1.67–5.91]; *p* = .04) compared with LIP‐EXC (Figure 1A; cell culture from *n* = 5 animals per group), but iNOS expression did not differ between the groups (Figure 1B; cell culture from *n* = 5 animals per group). In macrophages from adipose tissue, both IL‐6 and iNOS expression did not differ between the groups (Figure 1C,D; cell culture from *n* = 5 animals per group).

**FIGURE 2:**
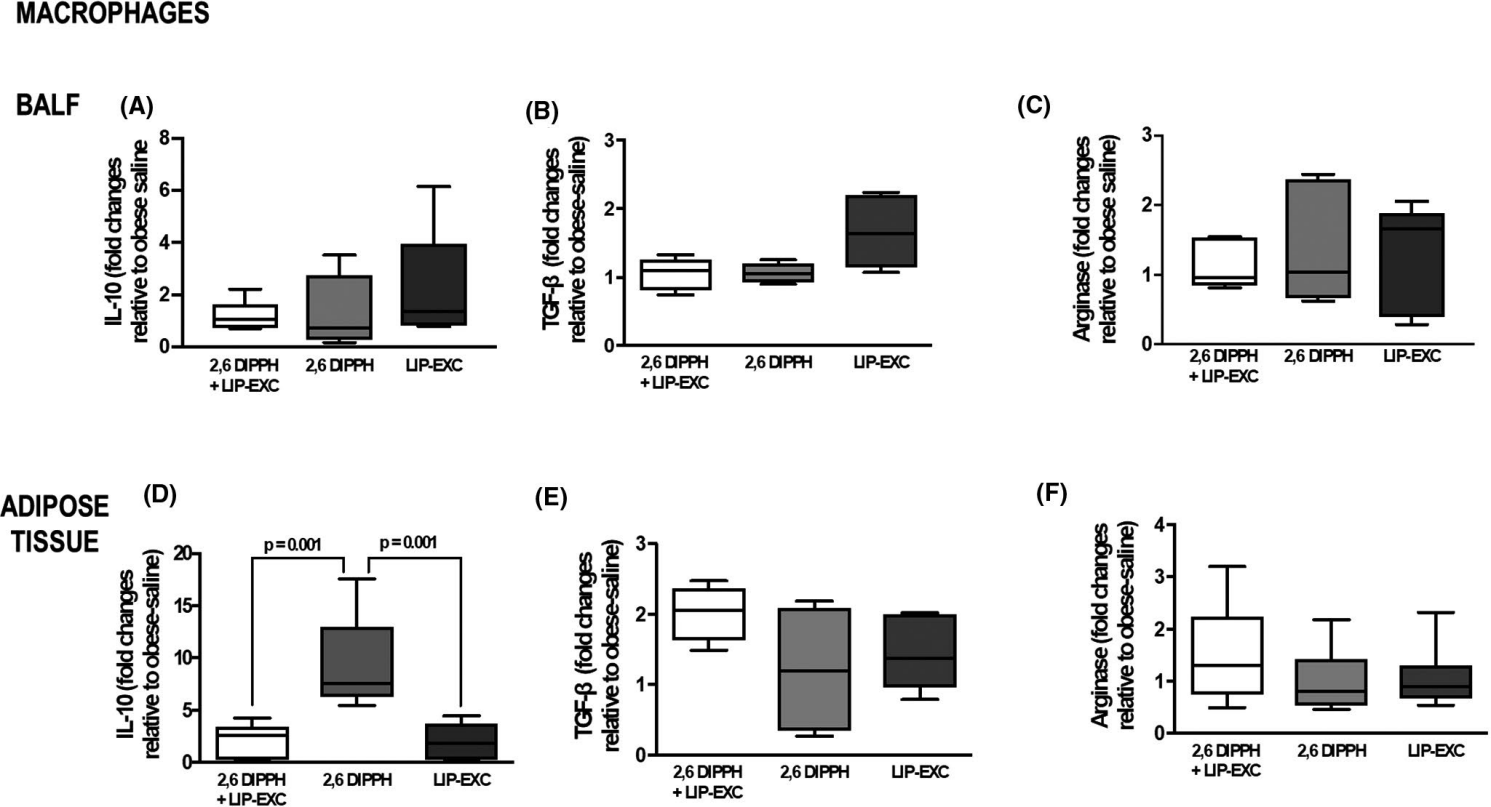
Alveolar and adipose tissue macrophages from obese animals (*n* = 5). Gene expression of interleukin (IL)‐10 (A, D); transforming growth factor beta (TGF‐β; B, E); and arginase (C, F) was assessed by reverse transcription polymerase chain reaction in macrophages stimulated for 1 h with the propofol composition (2,6‐diisopropylphenol [2,6‐DIPPH] +lipid excipient [LIP‐EXC]), active propofol component (2,6‐DIPPH), or LIP‐EXC. Data represent relative gene expression calculated as a ratio of the average expression of the target gene compared with the reference gene *36B4* and expressed as fold change relative to unstimulated cells. Samples were measured in triplicate; values are depicted as box plots (median, interquartile range, and minimum and maximum). Comparisons between groups were performed using the Kruskal–Wallis test followed by Dunn's post‐hoc test. BALF, bronchoalveolar lavage fluid

In alveolar macrophages, M2 markers, such as IL‐10, TGF‐β, and arginase, did not differ between the groups (Figure 2A–C; cell culture from *n* = 5 animals per group). In macrophages from adipose tissue, IL‐10 expression was higher after therapy with the active propofol component (2,6‐DIPPH) (median [interquartile range], 7.59 [6.28–12.95]; *p* = .001) compared with the other groups (Figure 2D; cell culture from *n* = 5 obese animals per group). Expression of TGF‐β and arginase did not differ between the groups (Figure 2E,F; cell culture from *n* = 5 animals per group).

#### 3.1.2 Effects of propofol (2,6‐DIPPH + LIP‐EXC), the active propofol component (2,6‐DIPPH), and lipid excipient (LIP‐EXC) on lung and adipose tissue neutrophils, neutrophil migration, endothelial cell damage, and fibrogenesis

**FIGURE 3:**
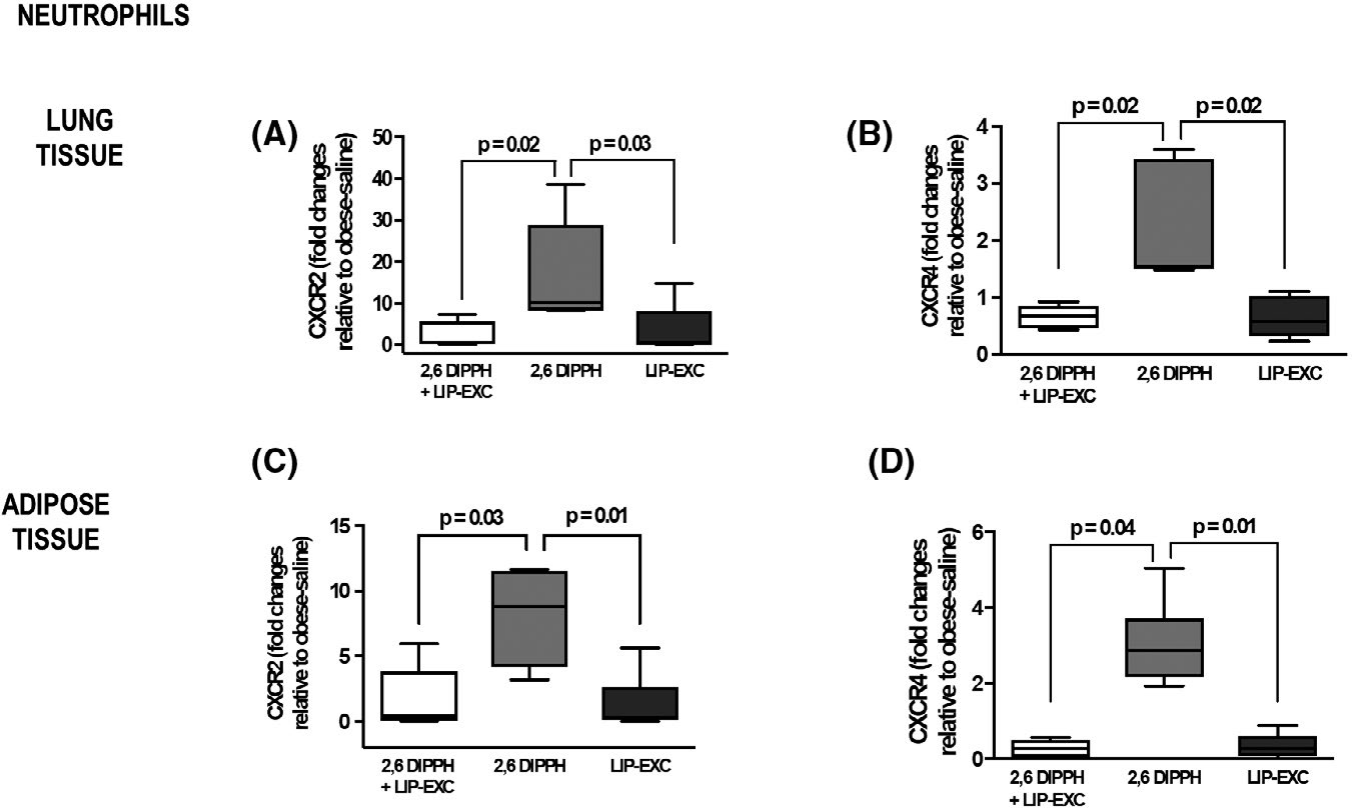
Neutrophils in lung tissue and adipose tissue from obese animals (*n* = 5). Gene expression of CXCR2 (A, C) and CXCR4 (B, D) was assessed by reverse transcription polymerase chain reaction in neutrophils stimulated for 1 h with the propofol composition (2,6‐diisopropylphenol [2,6‐DIPPH] + lipid excipient [LIP‐EXC]), active propofol component (2,6‐DIPPH), or (LIP‐EXC). Data represent relative gene expression calculated as a ratio of the average expression of the target gene compared with the reference gene *36B4* and expressed as fold change relative to unstimulated cells. Samples were measured in triplicate; values are depicted as box plots (median, interquartile range, and minimum and maximum). Comparisons between groups were performed using the Kruskal–Wallis test followed by Dunn's post‐hoc test

**FIGURE 4:**
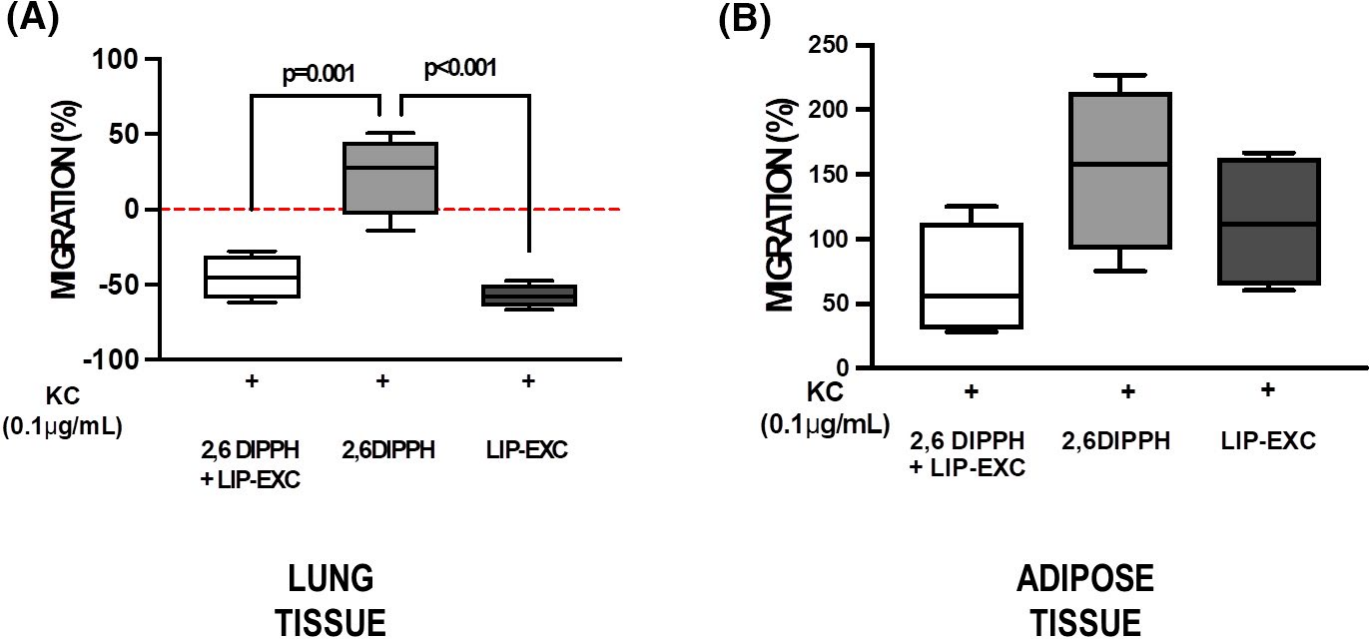
Percentage increase in neutrophil migration from lung (A) and adipose (B) tissue of obese animals (*n* = 4) stimulated for 1 h with the propofol composition (2,6‐diisopropylphenol [2,6‐DIPPH] +lipid excipient [LIP‐EXC]), active propofol component (2,6‐DIPPH), or LIP‐EXC in the top compartments of a chemotaxis chamber. Migrated neutrophils in response to cytokine‐induced neutrophil attracting chemokine (KC, 0.1 µg/ml) were counted after 60 min. Samples were measured in triplicate; values are the percentage variation related to saline control group cells (median, interquartile range, and minimum and maximum) of four animals/group

In both lung and adipose tissue, the expression of CXCR2 and CXCR4 was higher in the 2,6‐DIPPH group compared with the other groups (median [interquartile range], 10.08 [8.23–29.01]; *p* = .02; 8.78 [4.15–11.57]; *p* = .03; 1.55 [1.49–3.43]; *p* = .02; 2.86 [2.17–3.71]; *p* = .01, respectively) (Figure 3A–D, cell culture from *n* = 5 animals per group). In lung tissue, but not in adipose tissue, neutrophil migration was greater with active propofol component (2,6‐DIPPH) (median [interquartile range], 28.00 [−3.42 to 45.07]; *p* = .001) (Figure 4A,B; cell culture from *n* = 5 animals per group) compared with 2,6‐DIPPH + LIP‐EXC and LIP‐EXC. The expression of vascular cell adhesion molecules (VCAM‐1) in lung endothelial cells, PC‐I, PC‐III, and TGF‐β in lung fibroblasts did not differ between the groups (Figures S1 and S2).

### 3.2 Cells isolated from lean animals

#### 3.2.1 Effects of propofol, the active propofol component (2,6‐DIPPH), and lipid excipient (LIP‐EXC) on alveolar macrophages, lung tissue neutrophils, endothelial cells, and fibroblasts

The expression of IL‐6 and TGF‐β in alveolar macrophages, CXCR2 expression in lung tissue neutrophil, VCAM‐1 expression in lung endothelial cells, and PC‐III expression in lung tissue fibroblasts did not differ between the groups (Figure S3A–E; cell culture from *n* = 5 lean animals per group).

## 4 DISCUSSION

In obese animals, alveolar macrophages treated with propofol and its active component compared with lipid excipient showed higher expression of IL‐6. However, only propofol active component increased the gene expression of IL‐10 in adipose tissue‐derived macrophages, CXCR2 and CXCR4 in lung and adipose tissue neutrophils, as well as lung tissue neutrophil migration. Lung endothelial cells and fibroblasts were not affected by any of the tested agents. Cells from lean animals did not show any alterations after exposure to these agents for 1 h. In short, propofol composition affected macrophages and neutrophils in lung and adipose tissue in obesity but not in lean animals.

A diet‐induced obesity model, previously characterized by our group,^13^ presented increased body and trunk fat percentages, higher triglyceride, total cholesterol, and insulin and leptin levels compared with lean animals, as well as greater adipose tissue compartments normalized by body weight. This model was chosen since it resembles important aspects of human obesity, such as metabolic and hormonal changes.^13^ In addition, this obese model can elicit low‐grade inflammation as observed by increased levels of IL‐6 in lung and adipose tissues,^13^ suggesting that increased leptin levels may contribute to the release of pro‐inflammatory mediators through macrophages.^20^ Moreover, during obesity, immune cells may alter their receptor availability.^21^ Propofol, a widely used anesthetic agent, is composed of its active component and lipid excipient; the latter can change membrane structure and fluidity, leading to altered immune and inflammatory responses.^22^

In alveolar macrophages, IL‐6 expression was increased only with propofol and its active component, suggesting the lipid excipient had no significant impact. These results may corroborate pre‐clinical data^13^ supporting the hypothesis that increased leptin levels may contribute to the release of pro‐inflammatory mediators through macrophages.

In contrast, in adipose tissue macrophages, only the active propofol component increased gene expression of IL‐10, suggesting increased anti‐inflammatory M2 phenotype. These findings could suggest that the presence of the lipid excipient may counterbalance (or even abolish) the effect of the active component (2,6‐DIPPH) of propofol composition (2,6‐DIPPH + LIP‐EXC). Lipid emulsions, as used for the propofol vehicle, consist of a continuous aqueous phase (the glycerol content), triglycerides (long‐chain [LCT] or medium‐/long‐chain triglycerides), and emulsifiers (phospholipids, derived from egg yolk, the phosphatidylcholine component); emulsions also contain lipid‐soluble substances, such as vitamin E.^23^ The fatty acid structure of triglycerides, especially the chain length and the degree of unsaturation, is critical to the interaction of lipids with immune cells.^24^ The soybean oil structure, an LCT, is mainly linoleic acid, which is metabolized to arachidonic acid and generates bioactive lipid mediators,^25^ thus increasing oxidative stress.^26^ A possible trigger for the increase in IL‐10 is the transcription factor peroxisome proliferator‐activated receptor‐gamma,^27^ which may be increased by propofol exposure.^28^

Neutrophils are an essential component of the innate immune response and contribute to immune dysfunction in obesity.^29^, ^30^, ^31^ Neutrophil recruitment to the lung is primarily driven by blood neutrophil mobilization, mediated by cytokines including the CXC chemokines, which are increased in obesity.^32^ Neutrophil migration is regulated by chemokine receptors CXCR2 and CXCR4. Increased CXCR2 and decreased CXCR4 expression are associated with enhanced neutrophil release from the bone marrow and impaired homing toward the bone marrow.^33^ Expression of both CXCR2 and CXCR4 in neutrophils from lung and adipose tissues as well as neutrophil migration in lung tissue was higher after active propofol therapy compared with the other groups, which may also suggest the effects of lipid excipient hindering active effects of propofol. Neutrophil migration might be beneficial because it may contribute to defense mechanisms, such as bacterial clearance during an infection process, as well as restrict infection.^31^

Lung fibroblasts were isolated to investigate the contribution of the active propofol component or the lipid excipient on the modulation of fibrogenic factors. Activated lung fibroblasts, myofibroblasts, are capable of initiating remodeling by producing extracellular matrix (ECM) and inflammatory cytokines, which play pivotal roles in the pathogenesis of fibrosis.^34^ TGF‐β as well as procollagen I and III gene expression in fibroblasts did not differ between the groups in obesity, thus suggesting propofol acts more on inflammation than fibrogenesis.^35^

Obesity alters the expression of cell adhesion molecules VCAM‐1, ICAM‐1, and E‐selectin, modifying the expression of structural proteins such as pulmonary endothelial junctional adherens proteins (VE‐cadherin and β‐cathenin) by augmenting inflammation and disrupting the functions of the endothelial barrier.^36^ Although we did not find differences between the groups tested on expression of VCAM‐1, in the scenario of hypoxia‐reperfusion, immortalized cells submitted to the active ingredient (2,6‐DIPPH) and a commercial propofol formulation attenuated ICAM‐1 and VCAM‐1 expression, attributable to release of reactive oxygen species,^37^ and prevented p‐selectin increase.^38^ These protective actions decrease neutrophil influx, transendothelial migration, and neutrophil rolling/adhesion, which may decrease the severity of the early neutrophil component and tissue injury.

In cells from lean animals, no significant changes were observed between the groups, thus suggesting that the immune inflammatory response of obesity is necessary for the effects of active propofol and the lipid excipient.

This is the first study to evaluate propofol and its main formulation components separately considering the immunomodulatory properties in the scenario of low‐grade inflammation in obesity. Our results have shown diverse effects among the immune cells tested and their origin, which may represent different functions, but they show a trend toward an anti‐inflammatory profile on adipose tissue macrophages and neutrophils for the active components separately. As lipid emulsions are also known to present immunomodulatory effects,^39^ and they are a constitutive part of most available propofol compositions, it is important to understand their properties in the obesity environment. Propofol is largely used in the obese population. Obesity per se may have considerable impact on peri‐ or postoperative outcomes for individuals, but whether these findings may also translate into outcome‐relevant decision‐making relating to the choice of anesthetic agent for obese patients requires context‐sensitive and individualized appraisal.

### 4.1 Limitations

This study has some limitations: first, there is an important difference between human and murine neutrophils concerning chemokine receptor expression on the surface of neutrophils. Rodents do not express CXCL8, the major CXCR2 neutrophil ligand in humans. Second, the dose was chosen according to the clinical application, 50 μM propofol.^10^ Third, lipid excipients obtained from Cristália Laboratories may not have the same lipid components as the propofol purchased from another company. Therefore, further studies are required to evaluate the immunomodulatory effects of different propofol formulations, mainly in obese patients.

## 5 CONCLUSION

In the present *in vitro* study, in the obesity scenario, the active propofol component (2,6‐DIPPH) and the lipid excipient have different immunomodulatory effects, which results in diverse effects of the overall formulation on the cells tested. The active propofol component presented diverse immunomodulatory effects depending on the immune cell, its origin, and context.

## 6 ETHICS

This study was approved by the Ethics Committee for the Use of Animals (CEUA 020/16) of the Health Sciences Centre of the Federal University of Rio de Janeiro (UFRJ), Rio de Janeiro, Brazil.

## CONFLICT OF INTEREST

The authors have no conflict of interest.

## AUTHORS’ CONTRIBUTION

LH, FC, PP, PS, and PR contributed to study conceptualization, data analysis, interpretation, and manuscript preparation. LH, FC, MA, CB, LA, RL, and SA contributed to experimental conduct, methods, and data analysis. LH, FC, MA, and SC contributed to mRNA data analysis. LH, FC, PS, PP, and PR contributed to manuscript revision/finalization. All authors had access to the data and reviewed the manuscript.

## Supporting information

Fig S1‐S3

## Data Availability

The datasets presented in this study can be found in online repositories. The details on the repository/repositories and accession number(s) can be found in the Supplementary Information.
