# Supplementary material for: Effects of propofol and its formulation components on macrophages and neutrophils in obese and lean animals
Source: Pharmacol Res Perspect. 2021 Oct 10;9(5):e00873. doi: 10.1002/prp2.873 (PMC8503301; doi:10.1002/prp2.873)
Supplement: Supplementary file 1 — Fig S1‐S3 [file PRP2-9-e00873-s001.docx]

**Supplementary Information**

Effects of propofol and its formulation components on macrophages and neutrophils in obese and lean animals

Luciana Boavista Barros Heil,^1^ Fernanda Ferreira Cruz,1 Mariana Alves Antunes,^1^ Cassia Lisboa Braga,^1^ Lais Costa Agra,^1^ Rebecca Madureira Bose Leão,^1^ Soraia Carvalho Abreu,^1^ Paolo Pelosi,^2,3^ Pedro Leme Silva,^1^ Patricia Rieken Macedo Rocco^1^

^1^Laboratory of Pulmonary Investigation, Carlos Chagas Filho Institute of Biophysics, Federal University of Rio de Janeiro, Rio de Janeiro, Brazil

^2^Department of Surgical Sciences and Integrated Diagnostics, University of Genoa, Genoa, Italy

^3^Anesthesia and Intensive Care, San Martino Policlinico Hospital – IRCCS for Oncology and Neurosciences, University of Genoa, Genoa, Italy

SUPPLEMENTARY FIGURE 1 Lung endothelial cells from obese animals (*n* = 5). Gene expression of vascular cell adhesion molecule 1 (VCAM-1) was assessed by reverse transcription polymerase chain reaction in lung endothelial cells stimulated for 1 h with the propofol composition (2,6-diisopropylphenol [2,6-DIPPH] + lipid excipient [LIP-EXC]), active propofol component (2,6-DIPPH), or (LIP-EXC). Data represent relative gene expression calculated as a ratio of average expression of the target gene compared with the reference gene *36B4* and expressed as fold change relative to unstimulated cells. Samples were measured in triplicate; values are depicted as box plots (median, interquartile range, and minimum and maximum). Comparisons between groups were performed using the Kruskal-Wallis test followed by Dunn’s post-hoc test.

**** ****

**(C)**

**(B)**

**(A)**

SUPPLEMENTARY FIGURE 2 Lung fibroblasts from obese animals (*n* = 5). Gene expression of Procollagen I (PC-I) (A), Procollagen III (PC-III) (B), and transforming growth factor beta (TGF-β) (C) were assessed by reverse transcription polymerase chain reaction in lung fibroblasts stimulated for 1 h with propofol composition (2,6-diisopropylphenol [2,6-DIPPH] + lipid excipient [LIP-EXC]), active propofol component (2,6-DIPPH), or LIP-EXC. Data represent relative gene expression calculated as a ratio of the average expression of the target gene compared with the reference gene *36B4* and expressed as fold change relative to unstimulated cells. . Samples were measured in triplicate; values are depicted as box plots (median, interquartile range, and minimum and maximum). Comparisons between groups were performed using the Kruskal-Wallis test followed by Dunn’s post-hoc test.

**(B)**

**(A)**

**BALF Macrophages**

**(D)**

**(C)**

**Lung Tissue Neutrophils Lung Endothelial cells**

**(E)**

**Lung Tissue Fibroblasts**

SUPPLEMENTARY FIGURE 3 Cells isolated from lean animals (*n* = 5). Alveolar macrophages, gene expression of interleukin (IL)-6 (A) and transforming growth factor beta (TGF-β) (B) in bronchoalveolar lavage fluid (BALF); neutrophils in lung tissue, gene expression of CXCR2 (C); lung endothelial cells, gene expression of vascular cell adhesion molecule 1 (VCAM-1) (D); and lung fibroblasts, gene expression of Procollagen III (PC-III) (E) were assessed by reverse transcription polymerase chain reaction in each cell group stimulated for 1 h with propofol composition (2,6-diisopropylphenol [2,6-DIPPH] + lipid excipient [LIP-EXC]), active propofol component (2,6-DIPPH), and LIP-EXC. Data represent relative gene expression calculated as a ratio of the average expression of the target gene compared with the reference gene *36B4* and expressed as fold change relative to unstimulated cells. Samples were measured in triplicate; values are depicted as box plots (median, interquartile range, and minimum and maximum). Comparisons between groups were performed using the Kruskal-Wallis test followed by Dunn’s post-hoc test.
